# Posterior fossa tuberculoma in a Huichol native Mexican child: a case report

**DOI:** 10.1186/1756-0500-7-919

**Published:** 2014-12-16

**Authors:** Griselda Escobedo-Meléndez, Leopoldo Portillo-Gómez, Miguel A Andrade-Ramos, David Bocanegra, Rodrigo Mercado-Pimentel, Luis Arredondo, Dara Torres, Miguela A Caniza

**Affiliations:** Servicio de Hematología y Oncología Pediátrica, Unidad de Infectología, Hospital Civil de Guadalajara, Instituto de Investigación en Cáncer Infantil y de la Adolescencia, Universidad de Guadalajara, Guadalajara, Jalisco Mexico; Laboratorio de Microbiología y Parasitología, Departamento de Microbiología y Patología, Universidad de Guadalajara, Guadalajara, Jalisco Mexico; Servicio de Neurocirugía Pediátrica, Hospital Civil de Guadalajara, Guadalajara, Jalisco Mexico; Servicio de Anatomía Patológica, Hospital Civil de Guadalajara, Guadalajara, Jalisco Mexico; Servicio de Infectología Pediátrica, Hospital Civil de Guadalajara, Guadalajara, Jalisco Mexico; Department of Infectious Diseases, St. Jude Children’s Research Hospital, Memphis, TN USA; Unidad de Infectología, Servicio de Hematología y Oncología Pediátrica, Hospital Civil de Guadalajara “Dr. Juan I. Menchaca”, Salvador de Quevedo y Zubieta 750 Colonia Centro, Guadalajara, Jalisco 44340 Mexico

**Keywords:** Tuberculoma, Tuberculosis, Molecular diagnosis, Huichol child, Mexico

## Abstract

**Background:**

Tuberculosis is a major health concern in Mexico, especially among the native population. Tuberculomas are a frequent and severe complication of pediatric tuberculosis, these are observed as tumors in neuroimaging studies but are often not diagnosed adequately.

**Case presentation:**

We present a case of a 12-year-old native Mexican girl Huichol ethnicity diagnosed with a large posterior fossa tuberculoma found by imaging. This tuberculoma was surgically removed. Histopathologic examination and staining with hematoxylin and eosin, and Ziehl-Neelsen techniques of the surgical specimen were performed. Cerebrospinal fluid was analyzed by using the newly available Xpert® MTB/RIF assay (Cepheid, Sunnyvale CA, USA). Granulomatous inflammation with central caseous necrosis surrounded by edematous brain with reactive gliosis and acid-fast bacilli were revealed on histopathologic analysis. *Mycobacterium tuberculosis* DNA susceptible to rifampicin was detected in the patient’s cerebrospinal fluid and the patient was started on anti-tuberculosis treatment. The girl continued to show severe neurologic damage despite surgery and anti-tuberculosis treatment, and she eventually died of respiratory complications.

**Conclusion:**

Our case highlights the need for early confirmation of tuberculoma diagnosis by molecular assay so that timely treatment can be initiated to prevent severe brain damage. Furthermore, it emphasizes the need to consider tuberculomas in the differential diagnosis of children with neurologic symptoms living in areas of high tuberculosis incidence and those belonging to native populations in developing countries.

## Background

Pediatric tuberculosis (TB) is a global health threat [1], and tuberculomas are a frequent, severe complication of TB in developing countries [2, 3]. Intracranial tuberculomas occur in as many as 13% of children with neurotuberculosis [4]; these are observed as tumors in neuroimaging studies [5] but are often not detected at early stages, when antitubercular therapy offers a high likelihood of cure with minimal sequelae. Early diagnosis of tuberculomas can be difficult; they generally remain undetectable until they have grown sufficiently to cause neurologic signs and symptoms. Even a tumor detected by imaging cannot necessarily be attributed to TB because of frequent false-negative tuberculin skin tests (TSTs), and the difficulty of microscopic detection of TB in cerebrospinal fluid (CSF) and the slow growth of the bacilli in culture media [1, 2]. TB is a major health concern in Mexico [6], especially among the native population [7]. We report the unusual case of a native Huichol Mexican girl with a large posterior fossa tuberculoma; her TB was diagnosed by using the Xpert MTB/RIF assay, which amplifies DNA from *Mycobacterium tuberculosis* and detects rifampin resistance. The case presentation was approved by the Ethics Committee in Biomedical Research of Hospital Civil de Guadalajara.

## Case presentation

A 12-year-old Huichol native Mexican girl was admitted to the infectious disease ward of Hospital Civil de Guadalajara on February 3, 2012. She had been brought in a stretcher by her grandparents, who were her care providers. She had a history of headache, nausea, vomiting, blurred vision, weakness, and ataxia. Four months before admission she had begun complaining of headaches, which became progressively more intense, and occasional nausea and vomiting. One month before admission she experienced blurred vision, progressive weakness, and difficulty in walking and doing other voluntary movement. She had no fever or cough. A physician at the local healthcare center referred her to Hospital Civil. Soon after referral she began experiencing brief, generalized tonic-clonic seizures then, became too weak to walk.

The patient had not received routine childhood immunizations, including BCG vaccine. Her household provided poor living conditions, and her diet included unpasteurized cow’s milk. At examination at the hospital, the patient appeared emaciated: weight, 26 kg; height, 150 cm; body mass index (BMI), 11.6 (reference BMI for age, 18.5 – 24.9, by http://www.nhlbi.nih.gov/guidelines/obesity/BMI/bmi-m.htm, accessed May 2, 2014). Her heart rate was 121/min, respiratory rate was 60/min, blood pressure was 70/40 mm Hg, and axillary temperature was 36.3°C. She was unconscious and unresponsive to verbal commands. The patient showed constricted pupils unresponsive to light with bilateral convergent esotropia, generalized weakness, and bilateral flexor plantar responses.

**Figure 1:**
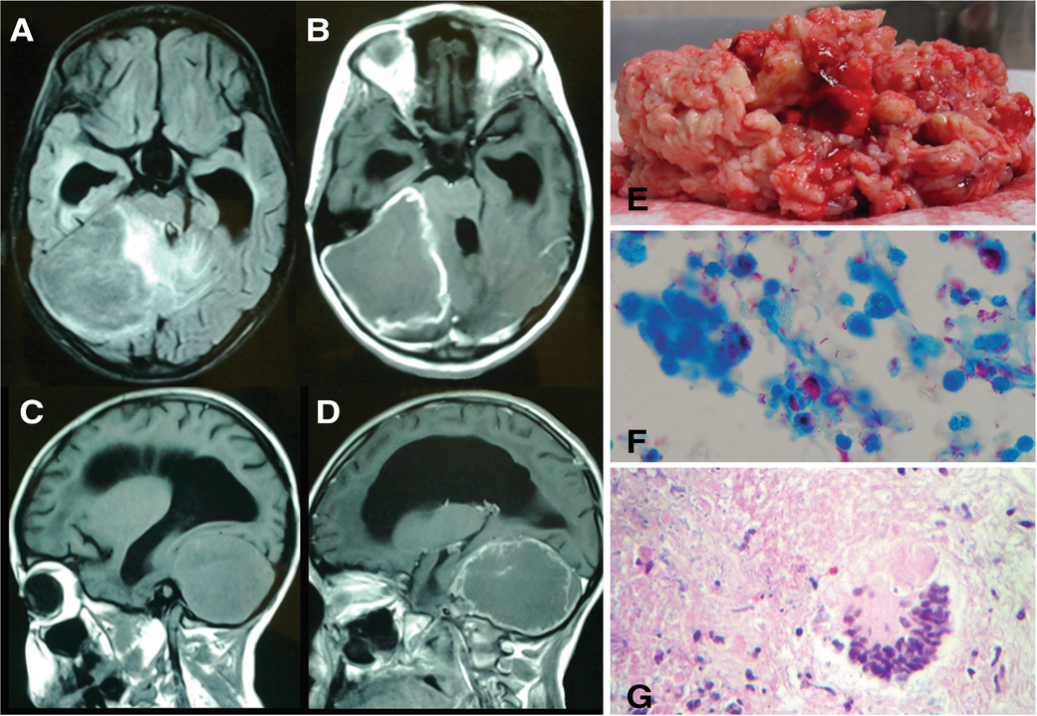
**Cranial magnetic resonance imaging of the tuberculoma and histopathologic studies of the surgical specimen. (A-B)** T1-weighted and contrast-enhanced T1-weighted axial MRI revealed a large (61 × 69 × 62 mm) heterogeneous lesion that was contralaterally displacing the fourth ventricle and pons. **(C-D)** Sagittal views show an intensely gadolinium-enhanced lesion occupying the entire posterior fossa and causing hydrocephalus by compressing the fourth ventricle. **(E)** The surgical specimen comprised a caseous-appearing material. **(F)** Acid-fast bacilli observed by Ziehl–Neelsen staining (original magnification, ×100). **(G)** Lymphoplasmacytic inflammation containing epithelioid cells and multinucleated Langhans giant cells forming a granulomatous lesion. Hematoxylin and eosin staining (original magnification, ×10).

Cranial axial computed tomography (CT) with contrast revealed a posterior fossa mass and hydrocephalus, confirmed by cranial magnetic resonance imaging (MRI) with gadolinium. T1-weighted MRI revealed a large, well-defined, heterogeneous mass in the right posterior fossa with hyperdense rim enhancement and hydrocephalus measuring 61 × 69 × 62 mm^3^ (Figure 1A-D). The lesion was initially interpreted as a primary brain tumor obstructing CSF circulation. A ventriculo-peritoneal shunt was immediately placed. The patient received intravenous mannitol 2 g/kg to reduce presumed cerebral edema. A few days later, a spinal T1-weighted MRI revealed marked gadolinium enhancement of the cervical, thoracic, and lumbar dura, suggestive of an infectious process such as TB. However, the TST was negative and the CSF contained no leukocytes. The CSF protein concentration was 230 mg/dL; the CSF glucose (37 mg/dL) and plasma glucose (113 mg/dL) ratio was 0.32 (reference range, >0.6). Acid-fast bacilli (AFB) staining by the Ziehl–Neelsen technique and culture were negative.

Because of the diagnostic uncertainty, the tumor was removed. It was revealed as a large, cheese-like mass (Figure 1E). Histopathology, using hematoxylin-eosin staining and Ziehl–Neelsen staining, revealed granulomatous inflammation with central caseous necrosis surrounded by edematous brain tissue with reactive gliosis and numerous AFB (Figure 1F-G). Rifampicin-susceptible *M. tuberculosis* was detected in the CSF by using the newly available Xpert MTB/RIF assay*,* confirming the diagnosis of central nervous system (CNS) TB. A chest CT, 10 days after admission, revealed consolidation in the basal left lung, but Ziehl–Neelsen staining and TB DNA amplification of bronchial and gastric aspirates with the Xpert MTB/RIF assay were negative for *M. tuberculosis*. Although her grandparents did not exhibit symptoms of TB, her community health department was notified to test other family members for TB (results not available).

The patient begun on 10 mg/kg/day isoniazid, 20 mg/kg/day rifampicin, 35 mg/kg/day pyrazinamide, and 20 mg/kg/day ethambutol. In addition, dexamethasone was added for the treatment of CNS TB. Serology was negative for human deficiency virus infection. One month after surgery and start of anti-TB treatment, the patient remained neurologically unchanged: unconscious, pupils unresponsive to light, bilateral esotropia, generalized weakness, and bilateral flexor plantar response. She was unable to communicate and was fed via orogastric tube. A cranial CT scan found no residual or new lesions or contrast enhancement at the original site.

The patient was cared for by team of infectious disease and multispecialty experts at Hospital Civil and medical care and medications were free. After three months, the grandparents requested a transfer to a hospital closer to her community. Six months after transfer, the patient died of respiratory complications. The Ethics Committee in Biomedical Research of Hospital Civil de Guadalajara granted permission and approval to review the patient’s clinical information.

## Discussion

In Mexico, native ethnic groups comprise 10% of the population [8] and have a higher incidence of TB [7] for reasons including genetic predisposition, lifestyle, limited healthcare access, and poverty [9]. The case we present is unusually advanced, even in such a medically underserved population and illustrates how healthcare deficiencies can affect children in low-income populations, such as late access to appropriate medical care and TB prevention. At the same time, it demonstrates the importance of a rapid, highly sensitive and specific diagnostic test for neurotuberculosis.

Our patient was severely malnourished, suggesting a long evolution of the disease. Thus, malnutrition, and consequently her immunocompromised condition, may explain her anergy to the TST. False-negative tuberculin reactions occur frequently in patients with advanced malnutrition [1, 2]. Our patient’s extent of neurological damage also indicated a long disease evolution. Her negative TST and the tuberculoma’s site may have delayed clinical suspicion until the CSF obstruction caused hydrocephalous [1]. Therefore, tuberculomas should be considered in the differential diagnosis of brain tumors in children living in TB-prone areas and in high-risk groups such as native populations in developing countries.

The patient’s neurologic symptoms evolved, in the absence of fever, for 4 months before hospitalization, consistent with the reported development of brain tuberculomas 2 to 6 months after TB infection [1]. Tuberculomas are granulomatous tumor-like mass that is not a cancer that results from infection with Mycobacterium tuberculosis. This lesion is composed of a central zone of caseation surrounded by collagenous tissue capsule that usually develop in the CNS or lungs [4, 5]. After hematogenous dissemination, the bacilli multiply locally and produce pathologic growth via chronic inflammation and aggregation of caseated granulomas [5]. A mass located in CNS structures frequently manifests as an intracranial space-occupying lesion [10, 11]. Therefore, tuberculoma should be a differencial diagnosis in patients with these intracranial lesions and severe neurologic damage where the risk TB is high, even in the absence of fever or other TB symptoms.

CT and MRI revealed a large mass in the posterior fossa, despite a negative TST and acid-fast CSF stain. Fortunately, the newly available MTB/RIF assay revealed TB. Despite its high sensitivity (100%) and specificity (85.7%) in diagnosing tuberculoma, CT has a low positive predictive value (33%) [10]. CT and MRI images are suggestive of malignant tumors, but MRI offers greater inherent sensitivity and specificity [11]. Moreover, traditional microbiologic tests for TB in the CSF have low sensitivity (26% to 37%) [1] and mycobacteria grow slowly in culture [2]. Our patient’s CSF stain was negative for AFB until subsequent testing by Xpert MTB/RIF amplification. AFBs were detected in the excised tuberculoma and the histopathologic studies demonstrated caseating granulomas and mycobacteria [1]. These studies are recommended in all suspected cases of TB similar to our case. The Xpert MBT/RIF kit has moderate sensitivity (59.3% to 81.3%) and high specificity (99.5% to 100%) in CSF specimens [12, 13]. It also rapidly detects *M. tuberculosis* in CSF and tissue specimens and should be compared with histopathologic findings [1, 5]. In Mexico, as in many countries in the Americas, this TB diagnostic test was introduced as part of the programmatic management of multidrug-resistant TB (http://www.paho.org/hq/index.php?option=com_docman&task=doc_view&gid=24329&Itemid=). This assay is recommended for use in CSF and tissue specimens by the World Health Organization guidelines for national pediatric tuberculosis programs [14].

Despite excision of the tuberculoma and treatment of the CSF obstruction and TB, our patient’s prognosis remained poor because of the tuberculoma’s size and neurologic sequelae. Her neurologic abnormalities were unremitting, and she died of respiratory complications, most likely related to chronic aspiration and infections. Risk factors for her poor outcome included the tuberculoma’s size, location, and lengthy evolution, and her malnourished condition. Other risk factors for an invasive TB and CNS infection is the lack of BCG vaccination, which has been reported to be protective for neurotuberculosis and miliary TB [1, 2]. Our case highlights the importance of BCG vaccination of children in TB-endemic regions and native ethnic groups in developing countries.

## Conclusion

Therefore, in areas with endemic TB, tuberculomas should be suspected in children with associated risk factors for TB infection [3, 5] and intracranial tumors. In clinically suspected cases, molecular assays now allow early diagnosis in CSF and tissues, facilitating timely treatment and prevention of severe brain damage and death.

## Patient consent

Written informed consent was obtained from the patient’s next to kin for publication of this Case Report and any accompanying images. A copy of the written consent is available for review by the Editor-in-Chief of this journal.

## Abbreviations

TB: Tuberculosis
TST: Tuberculin skin test
CSF: Cerebrospinal fluid
BMI: Body mass index
CT: Computed tomography
MRI: Magnetic resonance imaging
AFB: Acid-fast bacilli
CNS: Central nervous system
BCG: Bacillus calmette-guérin.
